# Chimeric Genes in Deletions and Duplications Associated with Intellectual Disability

**DOI:** 10.1155/2017/4798474

**Published:** 2017-05-24

**Authors:** Sonia Mayo, Sandra Monfort, Mónica Roselló, Carmen Orellana, Silvestre Oltra, Alfonso Caro-Llopis, Francisco Martínez

**Affiliations:** Unidad de Genética, Hospital Universitario y Politécnico La Fe, Avenida de Fernando Abril Martorell 106, 46026 Valencia, Spain

## Abstract

We report on three nonrelated patients with intellectual disability and CNVs that give rise to three new chimeric genes. All the genes forming these fusion transcripts may have an important role in central nervous system development and/or in gene expression regulation, and therefore not only their deletion or duplication but also the resulting chimeric gene may contribute to the phenotype of the patients. Deletions and duplications are usually pathogenic when affecting dose-sensitive genes. Alternatively, a chimeric gene may also be pathogenic by different gain-of-function mechanisms that are not restricted to dose-sensitive genes: the emergence of a new polypeptide that combines functional domains from two different genes, the deregulated expression of any coding sequence by the promoter region of a neighboring gene, and/or a putative dominant-negative effect due to the preservation of functional domains of partially truncated proteins. Fusion oncogenes are well known, but in other pathologies, the search for chimeric genes is disregarded. According to our findings, we hypothesize that the frequency of fusion transcripts may be much higher than suspected, and it should be taken into account in the array-CGH analyses of patients with intellectual disability.

## 1. Introduction

According to Kaye [1], chromosomal translocations and fusion oncogenes are the most frequent type of somatic DNA alteration in cancer, detected in 282 of the 384 validated cancer genes. Interstitial deletion can also generate fusion genes as described by Tomlins et al. [2] in prostate cancer [3], and although rare, fusion transcript has also been described due to tandem duplication as described by Jones et al. in pilocytic astrocytomas [4].

Chimeric genes can have different possible consequences: when due to a deletion, not only the loss of a fully functional copy of the gene is especially relevant for haploinsufficient genes, but also the gain-of-function of the chimeric gene. The new fusion protein might be pathogenic due to different mechanisms: the emergence of a new polypeptide that combines functional domains from two different genes, the deregulated expression of any coding sequence by the promoter region of a neighboring gene and/or a putative dominant-negative effect due to the preservation of functional domains of partially truncated proteins. The observed phenotype then may be a consequence of the CNV itself, but the chimeric gene might contribute and modify it, as in those cases with a wide spectrum phenotype associated to similar CNVs with different breakpoints.

Until now, few cases have been documented in the literature with intellectual disability (ID) due to fusion transcripts, all of them occurred de novo. Four were caused by chromosomal translocations that generated more than one fusion transcript [5–8], and another case was due to an interstitial deletion [9].

This paper describes three nonrelated patients with ID and different CNVs detected by array-CGH, generating different chimeric genes confirmed by different strategies. All the genes forming the fusion transcript might have an important role in the central nervous system (CNS) development and/or in gene expression regulation and therefore may contribute to the phenotype of both patients.

## 2. Material and Methods

The study was approved by the Ethical Committee on Clinical Research of the authors' hospital, and written informed consent was obtained from all participants. This research was carried out according to the principles of the Declaration of Helsinki. All positions in this study are based on the UCSC Genome Browser, National Center for Biotechnology Information (NCBI) build 37, hg19.

### 2.1. Sample Collection

Genomic DNA from the patients, their parents, and healthy controls was isolated from peripheral blood using QIAamp DNA Mini Kit and the QIAcube automated extractor (QIAGEN, Hilden, Germany). DNA quality and concentration were measured using the NanoDrop ND-1000 Spectrophotometer (NanoDrop Technologies, Rockland, DE, USA), and it was stored at −20°C.

Ficoll gradient centrifugation was used to isolate mononuclear cells from peripheral blood from the patients and healthy controls, following the manufacturer's recommendations (Lymphoprep AXIS-SHIELD PoCAS). Once purified, mononuclear cells were lysed in RLT buffer and total RNA was isolated using the RNasy kit (QIAGEN), as recommended by the manufacturer. RNA quality and concentration were measured also with the NanoDrop ND-1000 spectrophotometer. Total RNA was reversely transcribed in a final volume of 40 *μ*l, following the manufacturer's guidelines (Geneamp Gold RNA PCR Core Kit, Applied Biosystems, Foster City, CA, USA), and immediately stored at −80°C.

### 2.2. Microarray

Whole genome dosage analysis was performed by oligo-CGH-array (44K: G4426B Agilent Technologies, Palo Alto, CA, USA) and/or targeted custom array for ID and autism (manuscript in preparation). Array hybridization and scanning were performed following the manufacturer's specifications. The data were analyzed using the DNA analytics 4.0 software (Agilent Technologies).

### 2.3. Quantitative PCR Analysis

Real-time PCR assays were performed on the Light Cycler 480 (Roche, Basel, Switzerland). Reagents and programs of the PCR, as well as sequence and size of each amplicon, are available in Supplementary Table 1 available online at https://doi.org/10.1155/2017/4798474. Standard curves were generated from 2-fold serial dilutions of 60 ng female DNA. Reactions were done in triplicate, and melting curve analyses were performed to assess the specificity of the primers.

### 2.4. Characterization of the Breakpoints

**Figure 1:**
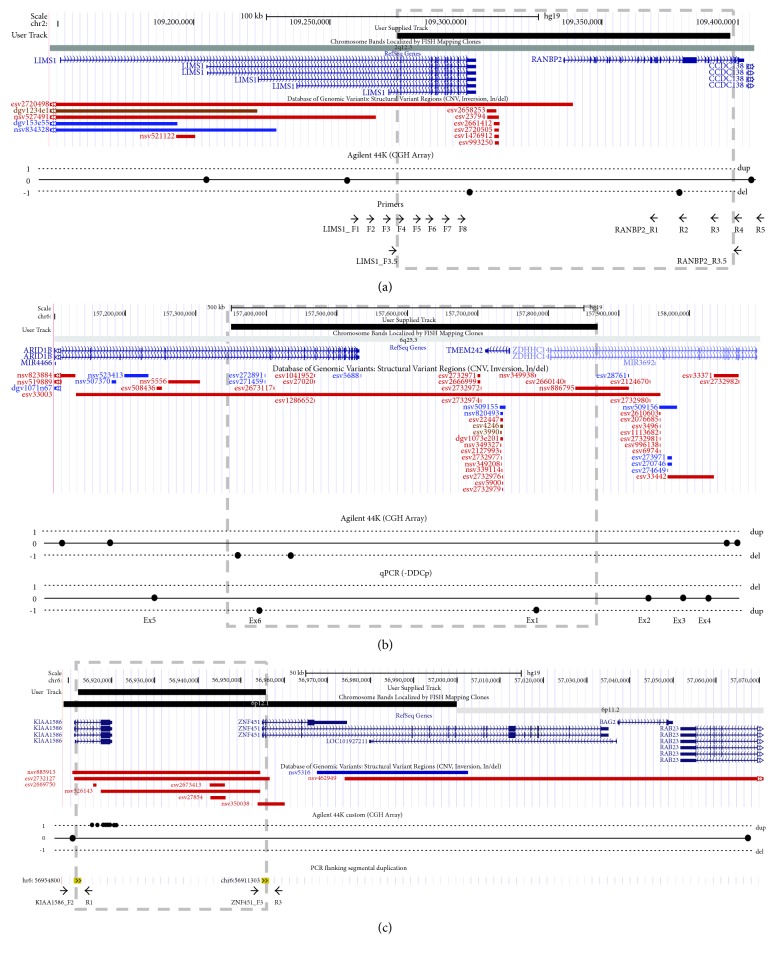
Representation of the CNVs in patient 1 (a), patient 2 (b), and patient 3 (c). CNVs are showed by a black box above and the grey dashed box. Refseq genes and the CNVs annotated in the region are indicated in the middle. Diagram of the array-CGH results and the strategies to detect the chimeric gene are showed below: the primers used to locate the breakpoint in patient 1 (a), the qPCR results in the exons of affected genes of patient 2 (b), and the primers flanking the segmental duplication in patient 3 (c).

A method based on Primer walking by long template PCR [10] was used to locate the breakpoint in patient 1. Starting from the array-CGH results, eight forward primers (LIMS1_F1-8) were designed in the 42 kb interval between the normal dosage probe and the deleted probe in *LIMS1* (chr2:109258450-109300532) and five reverse primers (RANBP2_R1-5) were designed in the 24 kb interval between the deleted probe and the normal dosage probe in *RANBP2* (chr2:109380702-109405259). The average spacing between primers was 5 kb (Figure 1(a)). PCR reactions for each forward-reverse pair of primers were processed on a PTC-200 thermocycler (BIO-RAD laboratories) (Supplementary Table 1).

### 2.5. Enzyme Digestion

A total of 8 *μ*l of the long template PCR product were digested 1 hour at 55°C with 1X digestion buffer 3 (New England Biolabs, Hitchin, UK) and 10U of BstXI (New England Biolabs) for patient 1. Fragment analysis was performed with a 12% polyacrylamide gel electrophoresis. According to the digestion pattern, a new set of primers was designed to amplify the approximately 1500 bp breakpoint-containing fragment with a specific PCR program (Supplementary Table 1, Figure 1(a)).

For patient 3, a total of 8 *μ*l of the long template PCR product (Supplementary Table 1) were double digested 1 hour at 37°C with 1X digestion buffer Tango (Fermentas, Burlington, Ontario, Canada) and 5 U of RsaI (Fermenta) and PstI (New England Biolabs). Fragment analysis was performed with a 12% polyacrylamide gel electrophoresis.

### 2.6. Fusion Transcript Detection

cDNA status was assessed by quantitative PCR using the constitutive gene glyceraldehyde-3-phosphate dehydrogenase (GAPDH) as control. Sequencing reaction and analyses were performed according to routine protocol on an ABI-3130XL Genetic Analyzer with the BigDye Terminator v1.1 Cycle Sequencing Kit (Applied Biosystems). Primer sequence, amplified size, and the PCR reagents and program are detailed in Supplementary Table 1.

### 2.7. Sequencing

Sequencing reaction and analyses were performed according to routine protocol on an ABI-3130XL Genetic Analyzer with the BigDye Terminator v1.1 Cycle Sequencing Kit (Applied Biosystems).

## 3. Results

### 3.1. Clinical Description

#### 3.1.1. Patient 1

The patient was the second-born child of healthy nonconsanguineous parents, a 29-year-old mother and a 34-year-old father. He was born to term by normal delivery after abortion risk in the first trimester. His birth weight was 3382 g (50th percentile), his length was 47 cm (25th percentile), and his head circumference was 33 cm (25–50th percentile). Neonatal malnutrition, gastroesophageal reflux, and persistent vomiting required one-month admission at hospital. Clinical examination at 21 months of age noted some dysmorphic features such as hypertrichosis, low anterior hairline, hypotelorism, downslanted palpebral fissures, epicanthus, anomalous teeth implantation, micrognathia, and dysmorphic, rotated, and low-set ears. His weight and height were in 3rd percentile, and he had microcephaly (<3rd percentile) and treated plagiocephaly. As congenital anomalies, he has aberrant origin of coronary artery and renal pyelectasis. He has mild intellectual disability and motor and speech development delay, he could not sit alone until the age of 14 months, and at the age of examination, he could not walk alone despite attending a centre for early developmental therapy and physiotherapy. He has axial hypotonia. In addition, he shows increased pain threshold, sound hypersensitivity, and anomalous behaviour such as avoidance of physical contact.

#### 3.1.2. Patient 2

The female patient was the first-born child of unrelated parents, a 25-year-old mother and a 23-year-old father. Her mother is healthy, and her father and paternal grandmother are diagnosed of bipolar disorder. She has a healthy brother. After 42 weeks of pregnancy, she was born by caesarean section due to lack of expansion. Her birth weight was 2740 g (10–25th percentile). She had neonatal eating disorders, and she did not cry until her first year of age. Clinical examination at 5 years of age noted some dysmorphic features such as microcephaly, round face, hypertrichosis, low anterior hairline, sparse hair with abundant hair fall, hypotelorism, synophrys, small and low-set ears, concave nasal ridge, thick lips, widely spaced teeth, and micrognathia. Her weight and height were low (3–10th percentile), and she has no significant congenital anomalies, apart from abnormal pigmentation of the skin, dystrophic toenails, clinodactyly, and short fingers. She has moderate intellectual disability with motor and speech delay. She could not walk until the age of 18 months, and at the age of examination, she has poor coordination and begins to talk with speech therapist's help. She attends a regular school with personalized support. She has no sleep disorder and is sociable.

#### 3.1.3. Patient 3

The female patient was born from unrelated healthy parents, a 22-year-old mother and a 27-year-old father. As family antecedents, her parents had two previous abortions and one child, who only lived 48 h, born after the patient. After 38 weeks of pregnancy, she was born by caesarean section due to lack of dilation. Her birth weight was 2750 g (25–50th percentile), and her length was 48 cm (25–50th percentile). She had neonatal feeding difficulties with an Apgar score of 6/9. Clinical examination at 5 years of age noted some dysmorphic features such as prominent forehead, big ears, hypotelorism, downslanted palpebral fissures, epicanthus, strabismus, depressed nasal root, anteverted nares, long philtrum, and malar flattening. As congenital anomalies, she presents agenesis of corpus callosum and an inguinal hernia. She has moderate intellectual disability and autism, with motor and speech delay. She could not walk until the age of 14 months, and at the age of examination, she has poor coordination and only speaks single words. She also has sleep disorder and aggressiveness towards others and herself.

### 3.2. Laboratory Findings

#### 3.2.1. Patient 1

The array-CGH showed deleted two contiguous probes, one located in *LIMS1* (A_14_P129225) and one located in *RANBP2* (A_14_P113906), two genes with the same orientation in chromosome 2 (2q12.3).

#### 3.2.2. Characterization of the Breakpoints

**Figure 2:**
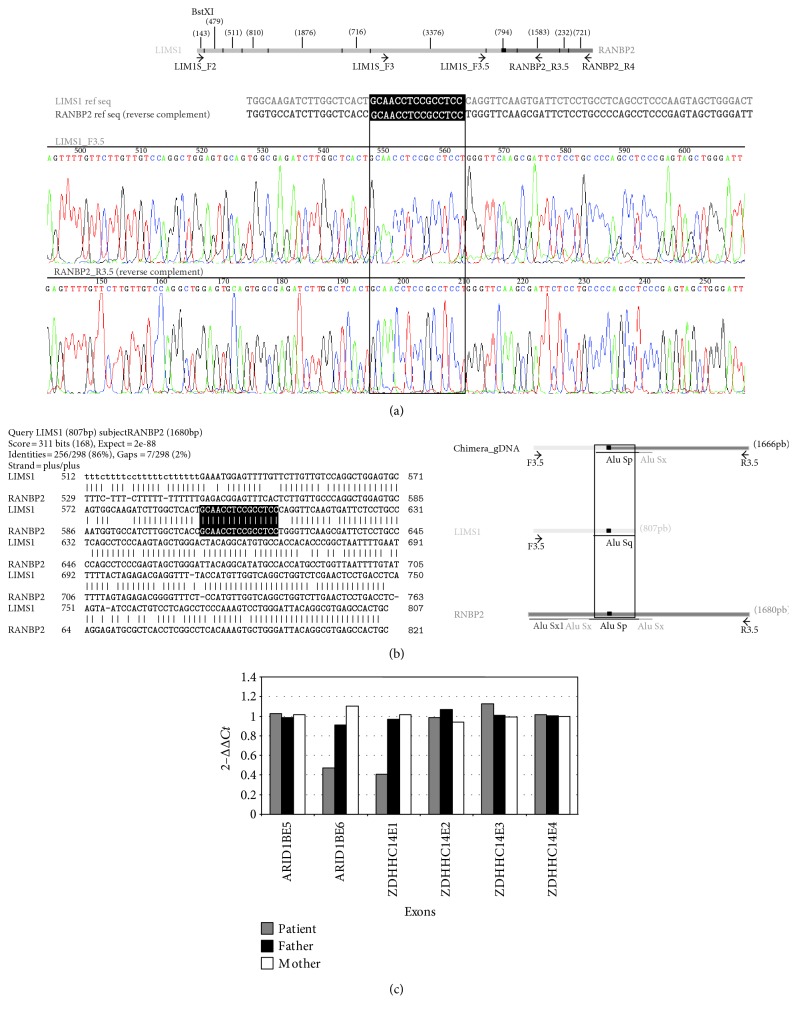
(a) Digestion pattern of LIMS1F2-RANBP2R4 amplicon with BstXI. The size of each fragment is showed in brackets, the black box represents the 15 nt match between both genes, and primers are indicated as black arrows (above). The sequence of the breakpoint is showed below with the 15 nt squared in white letters and in a black box in the reference sequence and in a black empty box in the sequencing sequence. (b) (Left) BLAST from *LIMS1* and *RANBP2* sequences at that region. The matched nucleotides are white in a black box. (Right) representation of the Alu elements contained in the breakpoint regions. The black box represents the 15 nt sequence showed left. (c) qPCR results of exons 5 and 6 of *ARID1B* gene and of exons 1, 2, 3, and 4 of *ZDHHC14* gene.

LIMS1F2-RANBP2R4 PCR and LIMS1F3-RANBP2R4 PCR showed specific bands for the patient, and not for his parents, of approximately 11 kb and 6 kb, respectively (data not show). The digestion pattern of the LIMS1F2-RANBP2R4 product PCR with BstXI allowed narrowing down the breakpoint interval. A new set of primers (LIMS1F3.5-RANBP2R3.5) yielded a 1666 bp amplicon specifically for the patient and not for his parents. The sequencing of this amplicon allowed to locate the breakpoint in *LIMS1* at chr2:109274856-109274870 and the breakpoint in *RANBP2* at chr2:109397243-109397257, corresponding to a 122.4 kb deletion. Both breakpoints are located in the same 15 nucleotide stretch present in two different Alu elements (Figures 2(a) and 2(b)).

#### 3.2.3. Identification of the Fusion Transcript

**Figure 3:**
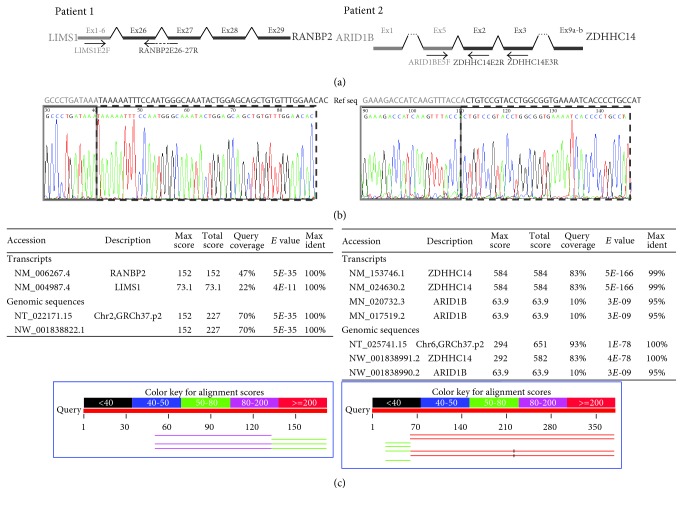
(a) Schematic representation of both chimeric genes in patients 1 and 2 and the primers used for its cDNA sequencing. (b) Sequence from RT-PCR cDNA, which confirm the presence of the fusion transcripts. A light-gray box underlines the 5′ end of the chimeric gene formed by *LIMS1* in patient 1 and *ARID1B* in patient 2, and a dark-grey dashed box indicates the 3′ end genes (*RANBP2* in patient 1 and *ZDHHC14* in patient 2). (c) BLAST from these sequences confirming that there are no other annotated regions in genome with such sequences (BLAST conditions: Database Human genomic plus transcript; optimize for highly similar sequences with a word size of 24).

According to the Refseq database, *LIMS1* encodes five different isoforms, all of them with 10 exons, where the last 9 exons are common (exon 7 to 15). The ten exons from all the isoforms are coding, except isoform b, which has two variants with alternative noncoding first exon (exons 2 and 3). *RANBP2* contains 29 coding exons. *LIMS1* is disrupted between the first exon of all isoforms and the second exon (exon 7), while *RANBP2* is disrupted between exons 25 and 26. Fusion transcript *LIMS1* (isoform b variant 2)-*RANBP2* was detected and confirmed by sequencing (Figure 3) in patient RNA but not in control RNA (specific primers and PCR conditions in Supplementary Table 1). The resulting chimeric transcript possess an open reading frame corresponding to exons 27 to 29 of RANBP2 giving a 260-aa hypothetical protein which contains the conserved domain of cyclophilin_ABH_like, implicated in protein folding processes.

#### 3.2.4. Patient 2

The array-CGH showed that two of the four probes located in *ARID1B* were deleted (A_14_P111041 and A_14_P106263). The next distal conserved probe was in *ZDHHC14*, which is orientated in the same direction and could also be partially deleted (region 5q25.3). The proximal breakpoint is contained in a 193 kb interval (chr6:157192799-157386210); while the distal breakpoint is located in a 623 kb interval (chr6:157454197-158076922). The big size of these regions did not allow locating precisely the breakpoints of the deletion by long template PCR, as in patient 1. In this case, a new strategy was performed. By qPCR analysis of exons 5 and 6 of *ARID1B* gene, and of exons 1, 2, 3, and 4 of *ZDHHC14* gene, we could refine the breakpoint locations between the exons 5 and 6 of *ARID1B* gene and between the exons 1 and 2 of *ZDHHC14* gene (Figure 1(b)). Both parents of the patient showed normal dosage for all the amplicons, confirming that the deletion occurred de novo in the patient (Figure 2(c)).

#### 3.2.5. Identification of the Fusion Transcript

According to the Refseq database, *ARID1B* gene encodes two different isoforms, in which exon 3 is alternatively spliced: isoform 2 with 20 exons (2249 aa) and isoform 1 with 19 exons (2236 aa). On the other hand, *ZDHHC14* gene, with 9 exons, encodes also for two different isoforms differing in the last exon. Fusion transcript between exon 5 of *ARID1B* and exon 2 of *ZDHHC14* was detected by RT-PCR in patient's RNA. This new transcript was absent in control RNA. Subsequent sequence analysis confirmed the presence of the fusion product (Figure 3). Primer sequence and technical information are provided in Supplementary Table 1.

Although the resulting chimera of *ARID1B* and *ZDHHC14* is not in the same reading frame, the transcript would codify for a 722-aa hypothetical protein. The first 679 amino acids coded by the 5′ region of *ARID1B*, while the 43 remaining amino acids of the C-terminal end would correspond to a new peptidic sequence.

#### 3.2.6. Patient 3

The custom array-CGH showed that 44 probes located in *KIAA1586* were duplicated. The proximal breakpoint is contained in a 4.1 kb interval within *KIAA1586* (chr6: 56911417-56915520), while the distal breakpoint is located in a 150 kb interval (chr6: 56920035-57070165) that contains four genes. A segmental duplication is located in both intervals (chr6:56911304-56913080 and chr6:56954801-56956578), and the distal one is contained in *ZNF451*, which is orientated in the same direction than *KIAA1586* (Figure 1(c)).

**Figure 4:**
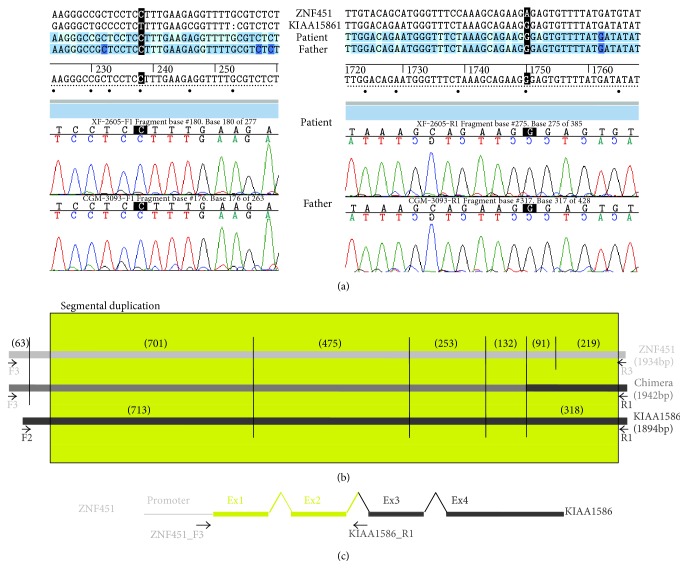
(a) Sequences from both ends of the ZNF451-KIAA1586 chimera. (b) Digestion pattern of PCR amplicons of ZNF451, KIAA1586 and the chimera with RsaI and PstI. The size of each fragment is showed in brackets, the yellow box represents the segmental duplication, and PCR's primers are indicated as black arrows. (c) Schematic representation of chimeric gene according to these results.

By PCR with specific primers of each region flanking the segmental duplication (ZNF451_F3-KIAA1586_R1), we could amplify the fusion fragment in the patient and her father. The sequence of both ends of the amplicon revealed that the 5′ region belongs to ZNF451 while the 3′ end is from KIAA1586. The digestion pattern of the PCR product with RsaI y PstI helped confirm the chimeric transcript (Figure 4).

The confirmation of the specific chimeric transcript in this case is not possible by RT-PCR due to the high homology of the sequence between the first exons of *ZNF451*and *KIAA1586* genes.

## 4. Discussion

We report the results of breakpoint analysis in three patients with intellectual disability associated with congenital anomalies carrying different chimeric genes.

The fusion transcript of patient 1 is generated from *LIMS1* first exon to *RANBP2* exon 26. The identification of the breakpoints inside Alu elements allows us to hypothesize that the fusion gene was generated by a nonallelic homologous recombination due to the high sequence homology (Figure 2).

*LIMS1* encodes for a highly conserved protein composed of five LIM domains arranged in tandem [11, 12]. Different studies support that LIMS1 participates in focal adhesion, signal transduction between the extracellular matrix and the intracellular network, and formation and maintenance of neuronal polarity [13–16]. On the other hand, RANBP2 is a component of the nuclear pore complex [17] with a wide range of functions, such as facilitation of protein traffic and sumoylation [18], energy maintenance in neurons [19–21], or chromosomal stability [22]. Some missense mutations in *RANBP2* are associated to acute necrotizing encephalopathy [23, 24]. Consequently, both genes have important but different roles in CNS development, so that their deletion may be pathogenic and contribute to the patient's phenotype. No copy number variations (CNV) comprising only these two genes are documented (Figure 1(a)). Although much larger deletions are recorded in different databases, which include total or partially *LIMS1* and *RANBP2* among other genes (DGV esv2720498; DECIPHER case 252497; and ISCA cases nssv579951), none of them are compatible with the generation of a chimeric gene.

**Figure 5:**
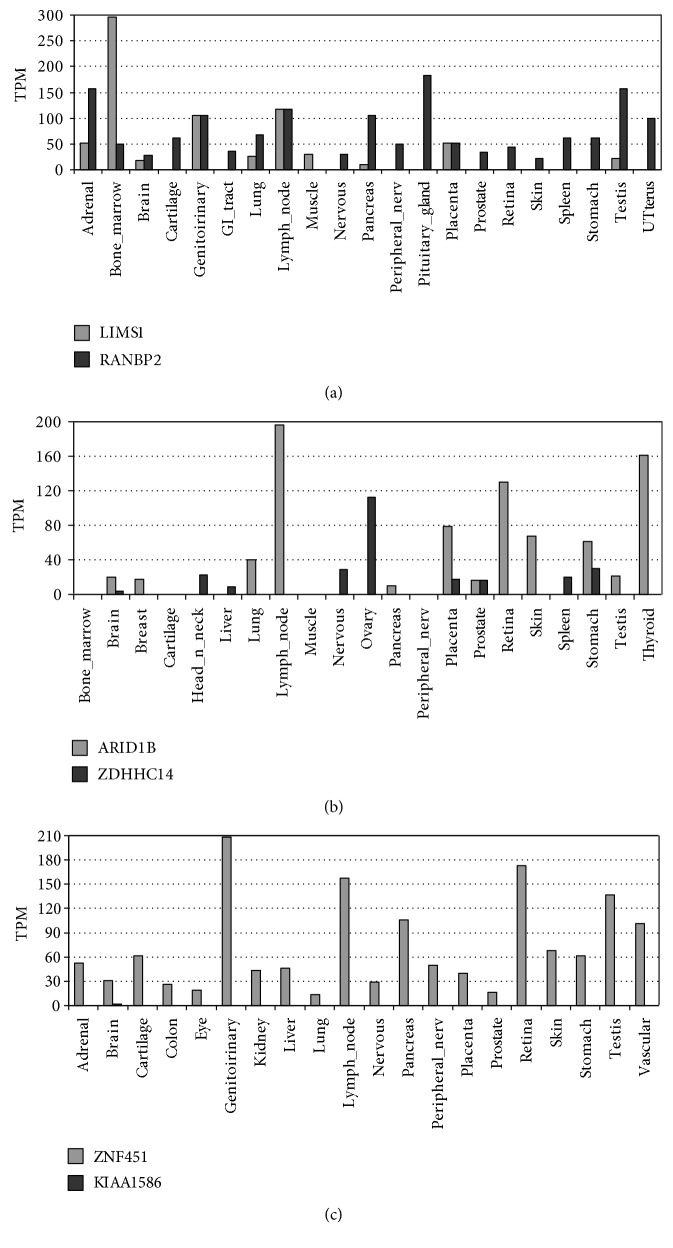
Differential expression pattern in the most relevant tissues from *LIMS1* and *RAMBP2* (a); *ARID1B* and *ZDHHC14* (b); and ZNF451and KIAA1586 (c) (GeneHub-GEPIS). TPM (transcript per million).

In addition to the effect due to haploinsufficiency of the respective genes, the fusion gene may also conceivably participate in the phenotype in different ways. The resulting chimeric transcript contains the conserved domain of cyclophilin_ABH_like, implicated in protein folding processes. The chimeric protein might interfere the function of RANBP2, by competing for its ligands, acting as a dominant-negative effector, by blocking ligands from RANBP2 or by generating a misfolding of them. Also, the expression of this new protein product would be deregulated, as it will be controlled from the promoter sequences (and transcription factors) of *LIMS1* gene, which has a different expression pattern (Figure 5(a)).

In relation with the chimeric gene of patient 2, it contains exons 1 to 5 of *ARID1B* gene and all the *ZDHHC14* exons, except the first one. ARID1B forms part of a family of proteins with DNA-binding capacity, implicated in the control of cell growth, differentiation, and development [25]. In contrast, not much is known about *ZDHHC14*, besides that it encodes for a zinc finger, and therefore is implicated in gene expression regulation.

*ARID1B* is associated with the Coffin-Siris syndrome [26–28], and the clinical features of the patient 2 are compatible with this diagnosis (mainly the neonatal eating disorders, ID, motor and speech delay, hypertrichosis, synophrys, dystrophic toenails, clinodactyly, and short fingers). Therefore, the haploinsufficiency of *ARID1B* explains these clinical signs in patient 2. However, this case also presents micrognathia, hypotelorism, and abnormal pigmentation of the skin, while she does not present other features associated to Coffin-Siris syndrome as corpus callosum abnormalities or hypotonia. This is consistent with the broad clinical variability associated to ARID1B mutations, which led Santen and Clayton-Smith [28] to propose that other genetic factors might modify the phenotype of *ARID1B* haploinsufficiency. In our case, this factor might well be the generation of fusion transcripts. In this regard, Backx et al. [7] described a patient with a balanced translocation t(6;14)(q25.3;q13.2), which generated reciprocal in-frame fusion transcripts of *ARID1B* and *MRPP3*, and presented intellectual disability and agenesis of corpus callosum.

Patient 3 is the only case we present with a chimeric gene due to an inherited duplication. As in patient 1, the fusion gene could be generated by a nonallelic homologous recombination mediated by a segmental duplication.

The chimera generated by the duplication contains the promoter region of *ZNF451* and most of the coding region of *KIAA1586* whereas the 5'UTR region and the first two coding exons included in the segmental duplication may be of either gene (Figure 2). Therefore, the expression of the chimeric gene, mainly constituted by *KIAA1586*, would be regulated by the promoter region of *ZNF45*, and since both genes have different expression patterns, the expression of this protein product would be deregulated.

Both *KIAA1586* and *ZNF451* encode for transcriptional factors with wide gene expression (Figure 5(c)). Bucan et al. [29] considered *KIAA1586* as an autism susceptibility gene, since it was deleted in 5 out of 1771 unrelated patients with autism spectrum disorders (ASD) and in none of the 2538 controls, although the same deletion can also be found in unaffected relatives (siblings and parents). Pinto et al. [30] considered *KIAA1586* as a candidate gene for ASD in the analysis for rare CNV (<1% frequency). However, the loss of one copy of *KIAA1586* mediated by this segmental duplication will always be accompanied by a significant deregulation of the expression of *ZNF451*, which would be under the control of the promoter of *KIAA1586* (Figure 1(c)). Since the expression patterns of both genes are very different, ectopic expression of this gene, not only the haploinsufficiency of *KIAA1586*, might well be responsible for the presumed association to autism.

In spite of the consequences of the deletion and the duplication mediated by the segmental duplication are quite different, the inherited duplication of patient 3 might also be a predisposing factor for neurodevelopmental disorder, as previously proposed for the deletion [30, 31].

In 2012 Holt et al. tried to systematically investigate the generation of fusion transcripts derived from rare CNVs associated with ASD in patients and controls [32]. Focusing on CNVs with a population frequency <1% and a size >30 kb, they estimated that 134 out of 2382 (5.6%) rare CNVs present in 889 ASD patients could lead to a fusion transcripts and they found a similar frequency to that in controls. They tested five selected duplications but only one fusion transcript was confirmed in affected and unaffected subjects. They concluded that there is no evidence that fusion genes generated by CNVs lead to ASD susceptibility. Evaluation of the generation of chimeric genes associated with pathologies as ASD and ID, with a low rate of recurrence and a large diversity of CNVs, is not easy, especially for duplications, which could be integrated in different *loci* or in different orientations. On the contrary, in our experience, focusing in deletions with breakpoints within regions of <315 kb in patients with ID and/or ASD, we estimated that 41 out of 68 (60%) deletions could lead to a fusion transcripts. All the selected cases tested, present in this paper, were confirmed, yielding a minimal proportion of 3% of deletions that generate new chimeric genes. Hopefully, new technologies will help in the identification of possible fusion transcripts generated by CNVs and a more precise estimation of its frequency.

Chimeric genes derived from CNVs have been occasionally described associated to other pathologies besides cancer [33, 34]. As far as we know, only five cases have been documented in the literature with intellectual disability associated with de novo fusion transcripts. Four of them were caused by chromosomal translocations generating two reciprocal fusion transcripts [5–8] and one due to an interstitial deletion similar to the cases we present in this work [9].

Therefore, this is the first report on chimeric genes generated by CNVs (microdeletions and microduplications) in three unrelated patients with intellectual disability.

## 5. Conclusions

Nowadays, array-CGH analyses are widely used in the study of ID and/or congenital anomalies, but only the loss or gain of genetic dosage is usually sought, and in many cases, the regions containing the breakpoints are not well defined. Theoretically, the occurrence or not of a chimeric gene may explain the different clinical consequences of similar microdeletions or microduplications, since the breakpoints could be different and so the consequences. Therefore, the possibility of generating a new chimeric gene, which may well be responsible or contribute to the phenotype observed, also should be taken into consideration in array CGH-analyses. We hypothesize that formation of fusion transcripts due to CNVs in ID patients may be a mechanism that should be taken into account in the array-CGH analyses from now on.

## Supplementary Material

Table S1: PCR reagents and programs, as well as sequence and size of each amplicon, if applicable, use in the different PCR reactions. All general reagents are from Roche except from Taq enzyme that is from Biotools.
