# Supplementary material for: Chimeric Genes in Deletions and Duplications Associated with Intellectual Disability
Source: Int J Genomics. 2017 May 24;2017:4798474. doi: 10.1155/2017/4798474 (PMC5463148; doi:10.1155/2017/4798474)
Supplement: Supplementary file 1 — Table S1: PCR reagents and programs, as well as sequence and size of each amplicon, if applicable, use in the different PCR reactions. All general reagents are from Roche except from Taq enzyme that is from Biotools. [file 4798474.f1.pdf]

Supplementary table

Table S1: PCR reagents and programs, as well as sequence and size of each amplicon, if applicable, use in the different PCR reactions. All general reagents are from Roche except from Taq enzyme that is from Biotools.

Primer	Sequence	PCR reagents	PCR program	Size (bp)	
LIMS1_F1	CCATTTGAGCAGGATAGAGGATG	1µl gDNA (100ng/µl)<br>1X PCR reac Buffer2<br>2.5U Taq Expand<br>480µM dNTPs<br>1µM Primer F<br>1µM Primer R<br>25µl Vf	3min 94ºC<br>30s 94ºC ⎤<br>30s 65ºC ⎬ 10 cycles<br>8min 68ºC ⎦<br>30s 94ºC ⎤<br>30s 65ºC ⎬ 20 cycles<br>8min 68ºC +20s/cy ⎦<br>7min 68ºC	Not applicable	Patient 1
LIMS1_F2	TAGGGAATTGAGGACCTCGCAC				
LIMS1_F3	GGTAGGGCTCCGAGGGGCTCGC				
LIMS1_F4	TCTAGAAATGCTGACCCCAGGC				
LIMS1_F5	GTGAGGGTATGCCATGAGCTAG				
LIMS1_F6	ATCAGTGAGGTGCTTCAGAAGC				
LIMS1_F7	GGACAGAAACAGCAAGCTCACC				
LIMS1_F8	GAAGCCAGTCTGTAAGAAGTGC				
RANBP2_R1	GAGCAGCTGTTACAATGCCACC				
RANBP2_R2	CAAGCCAGTCATGAAGCATGCC				
RANBP2_R3	CAGAATGGGGAGGACTATCAGC				
RANBP2_R4	TGGACTGTCCGCCTGTTCCATC				
RANBP2_R5	ATCCAAATCACCTGTCAACGAC				
LIMS1F3.5	GTGATGAACATGGTGTGTCTGC	1µl gDNA (100ng/µl)<br>1X PCR reac Buffer + Mg<br>2.5U Taq<br>240µM dNTPs<br>1µM Primer F<br>1µM Primer R<br>25µl Vf	3min 94ºC<br>1min 94ºC ⎤<br>30s 67ºC ⎬ 30 cycles<br>1min 72ºC ⎦<br>7min 72ºC	1666	
RANBP2R3.5	TCAAAAGTGTTTGCTACTGAAGAG				
LIMS1E2F	TCGCAGTGATGTGAAACTGG	1µl cDNA<br>1X PCR reac. Buffer + Mg<br>2.5U Taq<br>240µM dNTPs<br>500nM Primer F<br>500nM Primer R<br>50µl Vf	4min 94ºC<br>30s 94ºC ⎤<br>30s 55ºC ⎬ 36 cycles<br>30s 72ºC ⎦<br>7min 72ºC	208	
RANBP2E26-27R	TTACTTCTACCTCTGGTAGTG				
ARID1BE5F	CATGTCTCAGGAAGGCTATGG			426	Patient 2
ZDHHC14E3R	CGCAGTTATCACAAAGGCTGC				
ARID1BE5F	CATGTCTCAGGAAGGCTATGG			177	
ZDHHC14E2R	CAAAGAAGAACAGGATGCCAG				
ARID1BE5F	CATGTCTCAGGAAGGCTATGG	1µl gDNA (40ng/µl)<br>1X LC480 Probes Master mix<br>300nM Primer F<br>300nM Primer R<br>10µl Vf	10min 94ºC<br>10s 94ºC ⎤<br>10s 55ºC ⎬ 40 cycles<br>10s 72ºC ⎦<br>5s 95ºC<br>1min 65ºC<br>0.57ºC/s 97ºC	286	
ARID1BE5R	TATCCAGGACTACAGAGCTCC				
ARID1BE6F	GTCTGGCTCCATTGATGACC			235	
ARID1BE6R	GATACTTGCAGGAGAGATTGG				
ZDHHC14E1F	AAGACTGCGAGTACAGCCAG			218	
ZDHHC14E1R	CTCGAAGGCGAAGAAGAGTC				
ZDHHC14E2F	GTCTGTAAGACAGAACTGCAG			259	
ZDHHC14E2R	CAAAGAAGAACAGGATGCCAG				
ZDHHC14E3F	CACAGATATCGCAAACGGCAC			161	
ZDHHC14E3R	CGCAGTTATCACAAAGGCTGC				
ZDHHC14E4F	CTGCCTCTCATTTCAGAACGG			147	
ZDHHC14E4R	GACGTGGGTGATAACGAATGC				
KIAA1586_F2	TTCGCTGTAGCTCAGAGTC	1µl gDNA (100ng/µl)<br>1X PCR reac Buffer3<br>2.5U Taq Expand<br>480µM dNTPs<br>1µM Primer F<br>1µM Primer R<br>25µl Vf	3min 94ºC<br>30s 94ºC ⎤<br>30s 56ºC ⎬ 10 cycles<br>2min 68ºC ⎦<br>30s 94ºC ⎤<br>30s 56ºC ⎬ 22 cycles<br>2min 68ºC +20s/cy ⎦<br>7min 68ºC	1891	Patient 3
KIAA1586_R1	GAAACATTAGACAGACACTCC				
ZNF451_F3	CTTCGCTATCTCACAGGAC			1942	
KIAA1586_R1	GAAACATTAGACAGACACTCC				
ZNF451_F3	CTTCCCAGTGCTTCGCTATC			1934	
ZNF451_R3	TGCACATCTGACACTCAACG				
